# Relationship between intrahemispheric and interhemispheric connectivity of the language network and language improvement in subacute post-stroke aphasia

**DOI:** 10.3389/fneur.2025.1634902

**Published:** 2025-12-12

**Authors:** Xiaohui Xie, Yuqian Zhan, Mengdan Zhang, Kai Wang, Panpan Hu

**Affiliations:** 1 Department of Neurology, The Second Affiliated Hospital of Anhui Medical University, Hefei, China; 2 Department of Neurology, The First Affiliated Hospital of Anhui Medical University, Hefei, China; 3 Second Ward of Encephalopathy Hospital, Xi’an Hospital of Traditional Chinese Medicine, Xi’an, China; 4 School of Mental Health and Psychological Sciences, Anhui Medical University, Hefei, China; 5 Anhui Province Key Laboratory of Cognition and Neuropsychiatric Disorders, Hefei, China

**Keywords:** intrahemispheric and interhemispheric connectivity, resting-state functional magnetic resonance, language network, post-stroke aphasia, subacute

## Abstract

Speech production and comprehension are coordinated by a large-scale language network. The dynamic balance of intrahemispheric and interhemispheric connectivity within this network is essential for normal language processing. Stroke often significantly disrupts both the functional integrity and dynamic balance of the language network, leading to language deficits (aphasia). However, the brain’s adaptive potential to compensate for lesions in post-stroke aphasia (PSA) remains incompletely understood. A key unresolved question is whether recovery of language function in PSA is primarily facilitated by compensatory mechanisms within the left hemisphere, increased recruitment (“upregulation”) in the right hemisphere, or both. Building on prior research, we defined a language network encompassing canonical language areas. We employed resting-state functional magnetic resonance imaging (rs-fMRI) to quantify functional connectivity (FC) and investigated differences in intrahemispheric and interhemispheric connectivity within this network between 32 patients with PSA and 70 healthy controls (HCs). Furthermore, we examined the association between altered connectivity patterns at baseline and subsequent improvement in language function in the PSA group. Compared to the HCs, the patients with PSA exhibited increased intrahemispheric FC at baseline. Crucially, this increased intrahemispheric FC was positively correlated with the magnitude of language function improvement from baseline to follow-up. In addition, intrahemispheric FC was significantly higher than interhemispheric FC in the PSA group at baseline. These findings suggest that aberrant connectivity within the language network represents a neural substrate of language impairment in PSA and that heightened intrahemispheric connectivity within the residual left hemisphere language network may predict better recovery of language function in patients with subacute PSA. Collectively, network-based pathology analysis enhances our understanding of the neural mechanisms underlying both lesion effects and functional recovery in PSA.

## 1 Introduction

Language represents a higher-order cognitive function foundational to human communication, instantiated by large-scale, distributed neural architecture. This architecture is predominantly supported by a left-lateralized network of brain regions located in the frontal, temporal, and parietal cortices (1, 2). However, previous research has demonstrated that engagement of right-hemisphere homologs becomes increasingly significant with rising linguistic complexity (3–5), underscoring the principle of bilateral, dynamic cooperation for maintaining linguistic fluency. Therefore, disruption of this network integrity—resulting from insults such as focal lesions (6, 7) or neurodegenerative diseases (8–10)—clinically manifests as aphasia.

Post-stroke aphasia (PSA) manifests as a spectrum of heterogeneous language deficits arising from the stroke-induced disruption of structural and functional integrity within the language network. While classic lesion-symptom mapping studies have robustly implicated damage to cortical language areas as a primary driver of these impairments (11), the network-level consequences are crucial, which are vividly illustrated by non-invasive brain stimulation studies. For instance, applying continuous theta-burst stimulation (cTBS) to the left temporal lobe transiently increases error rates in picture-naming tasks, mimicking a focal cortical deficit (12). Furthermore, lesions extending to subcortical structures and their associated white matter tracts can sever the critical links required for sound-to-production mapping, thereby compromising the entire articulatory–phonological processing loop (13–15).

Disconnection between cortical and subcortical structures also contributes to aphasia (16, 17). Conductive aphasia is often attributed to arcuate fasciculus (AF) lesions that disconnect receptive and expressive language regions (18, 19). Similarly, primary progressive aphasia (20)—a neurodegenerative disorder group characterized by predominant language deficits—stems from large-scale network degeneration involving white matter pathway alterations. Beyond structural damage, abnormal functioning of the left hemisphere language network impairs language processing. Functional magnetic resonance imaging (fMRI) studies reveal reduced lateralization and activation in language production/processing areas in autism spectrum disorder (21, 22). Similarly, children with developmental language disorder or 22q11.2 deletion syndrome exhibit significantly reduced language-related activation during spoken language processing compared to typically developing peers (23). In summary, both structural and functional impairments within the left hemisphere language network can cause language deficits.

Although left hemisphere dominance for language is well-established, accumulating evidence implicates the right hemisphere in language processing (3, 24). Díaz et al. (25) demonstrated right-hemisphere activation during lexical-semantic processing in healthy participants. Consistent with this, patients with right-hemisphere infarction exhibited diverse language impairments (26). While left hemisphere facilitation of aphasia recovery is widely recognized, the role of the right hemisphere remains incompletely understood (27, 28). Some studies suggest that compensatory recruitment of right-hemisphere homologs aids language recovery (4), whereas others propose that increased right-hemisphere activity reflects release from transcallosal inhibition. Supporting the latter view, inhibitory low-frequency repetitive transcranial magnetic stimulation (rTMS) over the right inferior frontal gyrus improves picture-naming in chronic non-fluent aphasia (29, 30). Therefore, the right hemisphere contributes critically to both language execution and aphasia rehabilitation.

In summary, balanced interhemispheric connectivity is integral to uniquely human social communication (3, 24). Consistent with this view, Xu et al. (31) demonstrated that right-hemispheric recruitment increases with contextual complexity during task-based fMRI. These observations motivate a systematic investigation of intrahemispheric and interhemispheric coupling within the language network as a potential lever for optimizing PSA rehabilitation. However, the spatiotemporal dynamics of post-stroke connectivity reorganization are still incompletely defined.

Resting-state fMRI (rs-fMRI) (32) is an ideal modality for mapping intrinsic neural circuits by capturing spontaneous, low-frequency BOLD signal fluctuations (33). From these data, we can compute functional connectivity (FC), a measure of temporal coherence between remote brain regions, typically indexed by the Pearson correlation of their respective time courses (34). Indeed, functional connectivity, derived from various neurophysiological signals, has proven to be a robust method for quantitatively assessing diverse cognitive states, including mental fatigue and emotional responses (35–39). Our prior study in subacute PSA demonstrated network disruption, specifically reduced FC between the left inferior frontal gyrus (IFG) and both the bilateral supplementary motor areas and the right superior temporal gyrus (STG) (33). This aligns with findings in neurodegenerative conditions, where Montembeault et al. (40) observed decreased FC from the left anterior temporal lobe to bilateral language regions in semantic variant primary progressive aphasia relative to Alzheimer’s disease and cognitively unimpaired controls. Characterizing these connectivity patterns is fundamental to understanding large-scale network pathologies and the potential for functional restoration after brain injury.

This study investigates intrahemispheric and interhemispheric connectivity changes between residual language regions and their relationship with language improvement in subacute PSA. We hypothesize that PSA involves aberrant connectivity within the residual language network and that its reorganization predicts language recovery. This study will inform network-based rehabilitation strategies for PSA.

## 2 Materials and methods

### 2.1 Participants

A total of 32 aphasic patients with left hemispheric stroke were enrolled at the First Affiliated Hospital of Anhui Medical University (Hefei, China) (Supplementary Table S1), along with 70 age-, sex-, and education-matched healthy controls (HCs). Patients met the following inclusion criteria: (i) diagnosis of aphasia, (ii) age between 18 and 80 years, (iii) first-ever stroke, (iv) left hemisphere lesion, (v) right-handedness, and (vi) ≤ 3 months post-stroke onset. Both PSA patients and HCs were excluded if they had neurological/psychiatric disorders, head trauma history, or excessive head motion (>3 mm translation or >3° rotation). All 32 PSA patients underwent baseline fMRI and a language assessment (T1, <3 months post-stroke). A follow-up language assessment (T2) was conducted ≥4 weeks after T1. Due to various circumstances, 21 participants were lost to follow-up, ultimately leaving 11 patients who completed the T2 language assessment.

Written informed consent was obtained from all participants/guardians. The study adhered to the Declaration of Helsinki and was approved by the Anhui Medical University Ethics Committee (2019H009).

### 2.2 Clinical evaluation

All patients underwent assessments of language performance using the Aphasia Battery of Chinese (ABC) (41), a Chinese standardized variant of the Western Aphasia Battery. The ABC consists of four subsections: spontaneous speech, auditory comprehension, repetition, and naming. The total score is 100, with a score below 93.8 indicating aphasia.

### 2.3 Neuroimaging data acquisition

MR images were acquired on a 3.0-Tesla MR system (Discovery MR750, General Electric, Milwaukee, WI, United States). During the MRI scan, all participants were asked to close their eyes, remain awake, and not think about anything or move their bodies. All resting-state functional images were acquired using the following parameters: repetition time (TR) = 2,400 ms, flip angle = 90°, echo time (TE) = 30 ms, slice thickness = 3 mm, matrix size = 64 × 64, field of view = 192 × 192 mm^2^, and 46 continuous slices (one voxel = 3 × 3 × 3 mm^3^). We also obtained 188 T1-weighted anatomic images in sagittal orientation for each participant, with the following parameters: TR = 8.16 ms, TE = 3.18 ms, flip angle = 12°, field of view = 256 × 256 mm^2^, slice thickness = 1 mm, and voxel size = 1 × 1 × 1 mm^3^.

### 2.4 Resting-state fMRI preprocessing

Resting-state fMRI data were preprocessed using the Data Processing and Analysis for (Resting-State) Brain Imaging toolkit (DPABI) (42). The processing steps for each participant were as follows: First, the first five functional volumes were discarded to ensure magnetization stability. Next, slice-timing correction and head motion correction were applied to the remaining volumes. Individual anatomic images were co-registered to functional images. Subsequently, the functional images were spatially normalized to standard space using the DARTEL template, applied separately for each group. After spatial normalization, nuisance regression was performed using the 24 Friston motion parameters, white matter high signals, and cerebrospinal fluid signals as regressors. Finally, spatial smoothing (Gaussian kernel = 4 × 4 × 4 mm3) and a band-pass filter (0.01–0.1 Hz) were applied to the functional images.

### 2.5 Lesion overlap map

**Figure 1:**
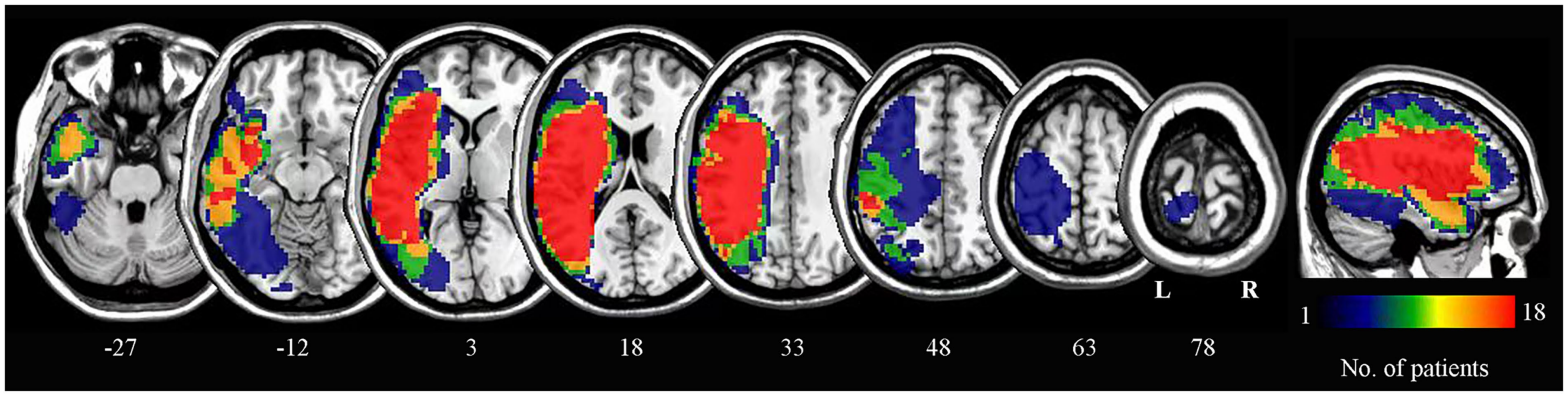
Lesion overlap map for all patients. The numbers below each axial slice correspond to z-plane coordinates in MNI space. The color scale indicates the number of patients with lesions in each voxel. L, left; R, right.

Lesion outlines were manually traced by a trained researcher on individual 3D T1-weighted structural images using the pen tool in MRIcron software to create a lesion mask for each patient. The transformation matrix derived from normalizing the individual 3D anatomical images to standard space was then applied to normalize each lesion mask to the same standard space. The normalized lesion masks from all patients were overlaid to construct the lesion overlap map (Figure 1).

### 2.6 Defining regions of interest of language areas

Given the left-lateralized nature of the language network, our analysis focused on perisylvian language regions, including the left inferior frontal gyrus (IFG), middle frontal gyrus (MFG), and superior temporal gyrus (STG). These areas encompass the classical Broca’s and Wernicke’s areas, which underlie language production and comprehension (6, 43). A total of eight regions of interest (ROIs) within these structures were selected to compute the language network (Table 1), consistent with prior validated methodologies for language network mapping (44).

**Table 1:** Regions of interest (ROIs) in the language network.

Hemisphere	Brain regions	MNI (x, y, z)
Left	IFG	−48, 30, −2
Left	IFG	−44, 25, −2
Left	IFG	−50, 19, 9
Left	MFG	−45, 14, 21
Left	STG	−54, −23, −3
Left	STG	−56, −33, 3
Left	STG	−48, −44, 3
Left	STG	−52, −54, 12

Montreal Neurological Institute (MNI) coordinates of peak activation points for the eight ROIs. IFG, inferior frontal gyrus; MFG, middle frontal gyrus; STG, superior temporal gyrus; ROI, region of interest.

### 2.7 Calculating the language network map in the healthy controls

**Figure 2:**
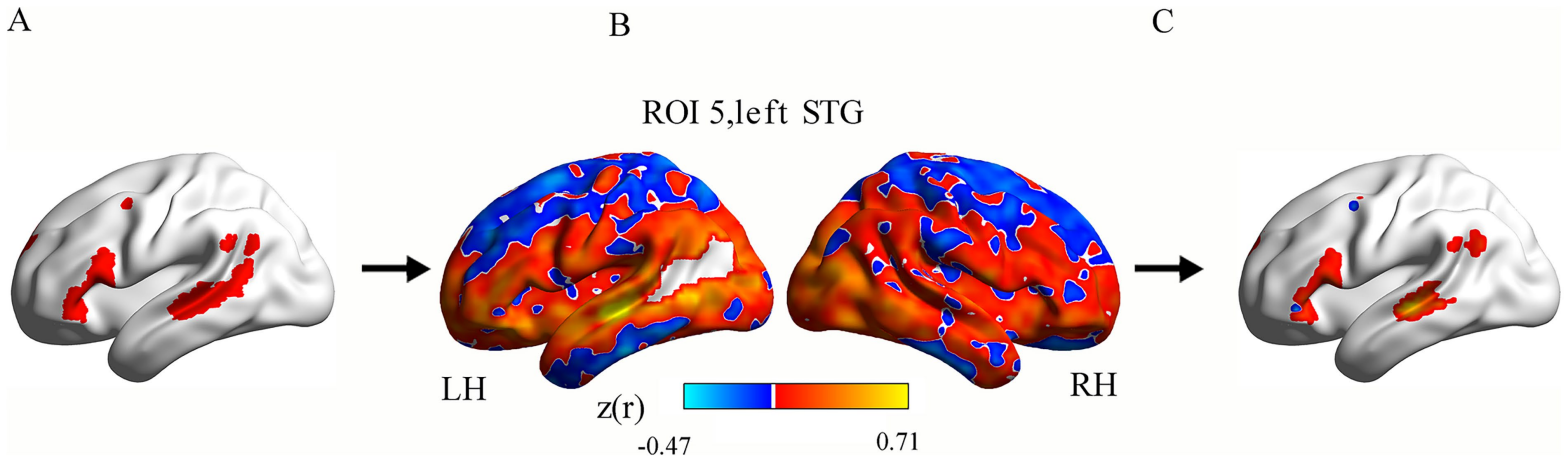
Calculation pipeline for intrahemispheric functional connectivity (FC) within the language network in a single patient. Panel **(A)** exhibits the left hemisphere language network (red). Panel **(B)** displays one patient’s ROI-based FC map for an ROI (e.g., left STG). Orange-yellow colors show voxels with positive FC with the ROI, while blue colors show negative FC. Panel **(C)** shows that each ROI-based FC map was masked using the language network template, producing ROI-masked FC maps. ROI, region of interest; FC, functional connectivity; STG, superior temporal gyrus; LH, left hemisphere; RH, right hemisphere.

In the cohort of 70 healthy controls, we derived a language network map using language-associated ROIs. This normative map served as a mask to extract intrahemispheric and interhemispheric functional connectivity (FC) values in the PSA patients. The computational methodology for FC quantification in this language network comprised the following steps: First, for each participant, region-specific FC maps were generated by computing Pearson’s correlation coefficients (r) between the time course of each ROI and all other voxels within the brain. Second, subject-level mean FC maps were generated by averaging FC maps across all ROIs. Subsequently, r-values in these mean maps were converted to z-values using Fisher’s r-to-z transformation. Finally, the group-level language network map was generated by averaging all subject-level mean FC maps. All the voxels with z(r) > 0.3 formed the final language network mask in the group-level FC map. This specific value is explicitly used and described in numerous high-impact neuroimaging studies (44, 45). Then, this language network mask was partitioned into left (Figure 2A) and right hemisphere (not shown) components for subsequent analyses.

### 2.8 ROI-based functional connectivity analysis in post-stroke aphasia

For each PSA patient, the following processing pipeline was implemented: First, Pearson’s correlation coefficients (r) were computed between each ROI’s time series and all voxels within the remaining brain (excluding lesioned areas). Fisher’s r-to-z transformation converted the coefficients to normalized z-values, generating eight ROI-based FC maps per participant. Second, each ROI-based FC map (Figure 2B) was masked using the language network template (derived from the HCs; voxel inclusion threshold: z(r) > 0.3; Figure 2A), producing ROI-masked FC maps (Figure 2C). Finally, we computed intrahemispheric FC as the mean z-value across voxels in the left hemisphere language mask for all ROI-masked FC maps, with interhemispheric FC similarly derived from the right-hemisphere language mask.

### 2.9 Statistical analysis

Independent samples *t*-tests were used to compare age and education years between the PSA patients and HCs. A chi-squared test compared sex distributions. For FC analyses, independent samples *t*-tests assessed intergroup (PSA vs. HC) differences in intrahemispheric and interhemispheric FC values, while Wilcoxon signed-rank tests evaluated intrahemispheric versus interhemispheric FC differences within the PSA group. Correlation analyses examined baseline functional variables versus language scores at T1 and T2. Partial correlations (controlling for time interval variability) quantified relationships between functional variables and language improvement (T2–T1). A significance threshold of *p* < 0.05 was used (two-tailed, uncorrected).

## 3 Results

### 3.1 Demographics and clinical characteristics

A total of 32 patients with PSA and 70 healthy controls (HCs) were enrolled. Demographic and clinical characteristics of the patients (mean age 55.22 ± 11.79 years) and HCs (mean age 54.63 ± 12.75 years) are summarized in Table 2. No significant differences were found in age, education years, or sex distribution between the groups. A total of 11 PSA patients (mean age 53.09 ± 12.02 years) completed follow-up language assessments. Significantly higher scores were observed at follow-up compared to baseline for the Aphasia Quotient (AQ) and all four subsections (Table 3).

**Table 2:** Demographic characteristics of the patients and healthy controls and clinical features of the patients.

Demographic indicators	PSA (*n* = 32)	HC (*n* = 70)	*p*-value
Sex (male/female)	31/1	65/5	0.73^a^
Age (years)	55.22 ± 11.79	54.63 ± 12.75	0.85^b^
Education (years)	9.06 ± 3.41	9.80 ± 3.44	0.38^b^
Disease duration (weeks)	4.00 ± 3.09	NA	NA
Lesion size (cm^3^)	42.41 ± 36.77	NA	NA
ABC scores			
Spontaneous speech^c^	8.59 ± 6.39	NA	NA
Auditory comprehension^c^	5.84 ± 2.45	NA	NA
Repetition^c^	3.34 ± 3.49	NA	NA
Naming^c^	2.26 ± 2.76	NA	NA
AQ^c^	40.07 ± 26.87	NA	NA

Values are presented as mean ± standard deviation; ^a^Chi-squared test between the healthy controls and patients at baseline; ^b^Independent samples t-test; ^c^Standardized scores according to the Western Aphasia Battery. PSA, post-stroke aphasia; HC, healthy controls; AQ, Aphasia Quotient.

**Table 3:** Comparison of language performance at baseline and follow-up in the patients.

Language performance (*n* = 11)	T1	T2	t/Z value	*p*-value
Spontaneous speech	7.91 ± 7.20	16.09 ± 3.83	3.839	0.003^b^
Auditory comprehension	6.39 ± 1.88	8.22 ± 1.64	4.247	0.002^b^
Repetition	3.09 ± 3.70	6.45 ± 3.60	−2.936	0.003^a^
Naming	1.80 ± 2.41	7.30 ± 3.97	4.841	0.001^b^
AQ	38.39 ± 28.42	73.19 ± 27.82	3.985	0.003^b^

T1, within 3 months of stroke onset; T2, at least 4 weeks after T1; AQ, Aphasia Quotient; ^a^Wilcoxon signed-rank test; ^b^paired samples t-test. *n* = 11 patients who completed language assessments at both T1 and T2.

### 3.2 FC analysis

**Figure 3:**
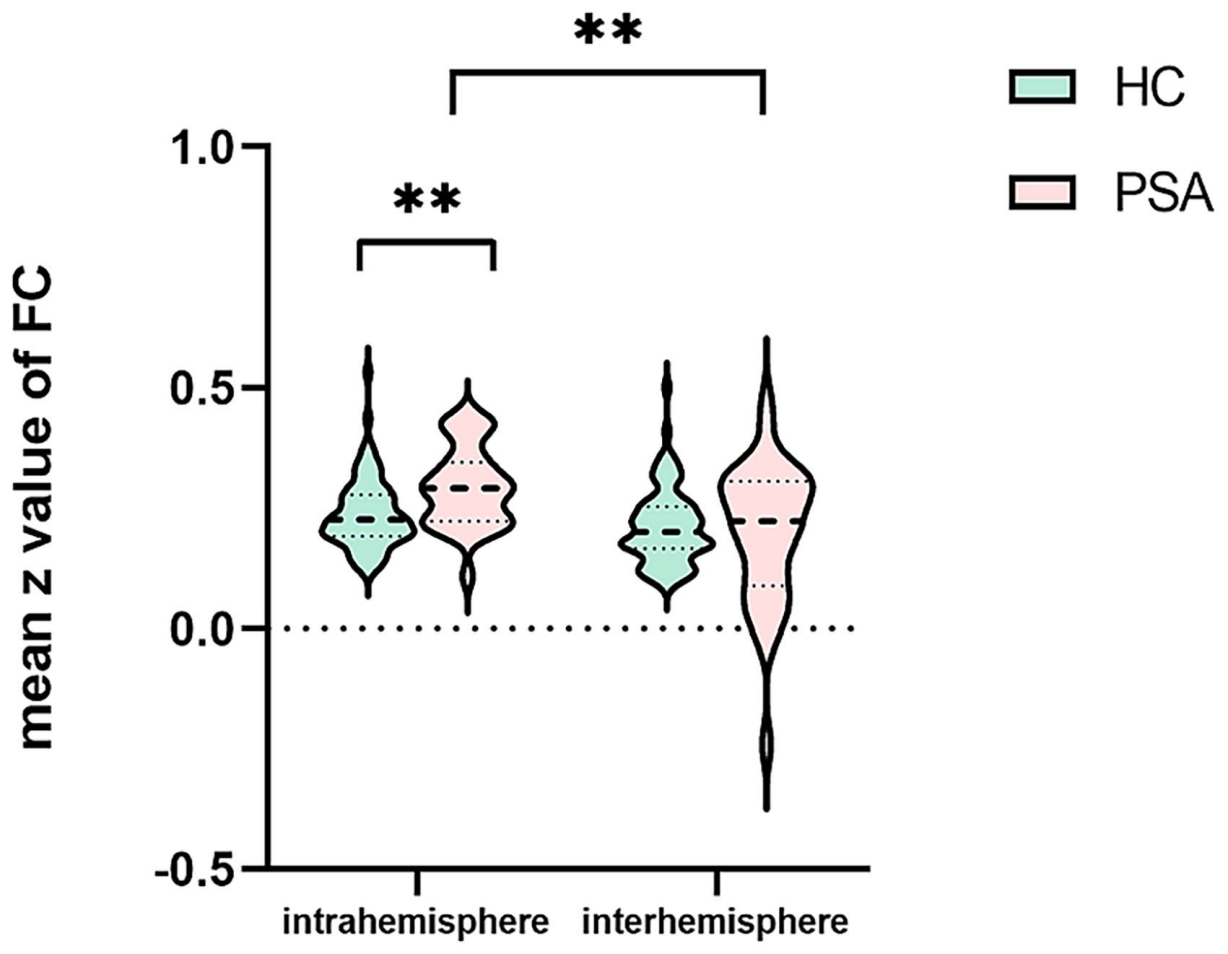
Intrahemispheric and interhemispheric functional connectivity (FC) in the patients with post-stroke aphasia (PSA) and healthy controls (HCs). ^**^p < 0.01.

Compared to the HCs, the PSA patients exhibited significantly increased intrahemispheric FC (t = 3.367, *p* < 0.01; Table 4; Figure 3). In addition, intrahemispheric FC was stronger than interhemispheric FC in both PSA patients and HCs (PSA: Z = − 3.902, *p* < 0.01; HC: t = 4.751, *p* < 0.01; Table 5; Figure 3). No significant intergroup differences emerged in interhemispheric FC (Table 4).

**Table 4:** Comparison of intrahemispheric and interhemispheric connectivity between the patients and healthy controls.

Functional connectivity	PSA (*n* = 32)	HC (*n* = 70)	t value	*p*-value
Intrahemispheric FC	0.29 ± 0.08	0.24 ± 0.07	3.367	0.001^a^
Interhemispheric FC	0.20 ± 0.14	0.21 ± 0.08	−0.477	0.636^a^

^a^Independent samples t-test. FC, functional connectivity; PSA, post-stroke aphasia; HC, healthy controls.

**Table 5:** Differences in intrahemispheric and interhemispheric connectivity within the patients and healthy controls.

Participants	Intrahemispheric FC	Interhemispheric FC	Z/t value	*p*-value
PSA (*n* = 32)	0.29 ± 0.08	0.20 ± 0.14	−3.902	<0.001^a^
HC (*n* = 70)	0.24 ± 0.07	0.21 ± 0.08	4.751	<0.001^b^

^a^Wilcoxon signed-rank test; ^b^paired samples t-test. FC, functional connectivity; PSA, post-stroke aphasia; HC, healthy controls.

### 3.3 Correlations of FC abnormalities with clinical symptoms

**Figure 4:**
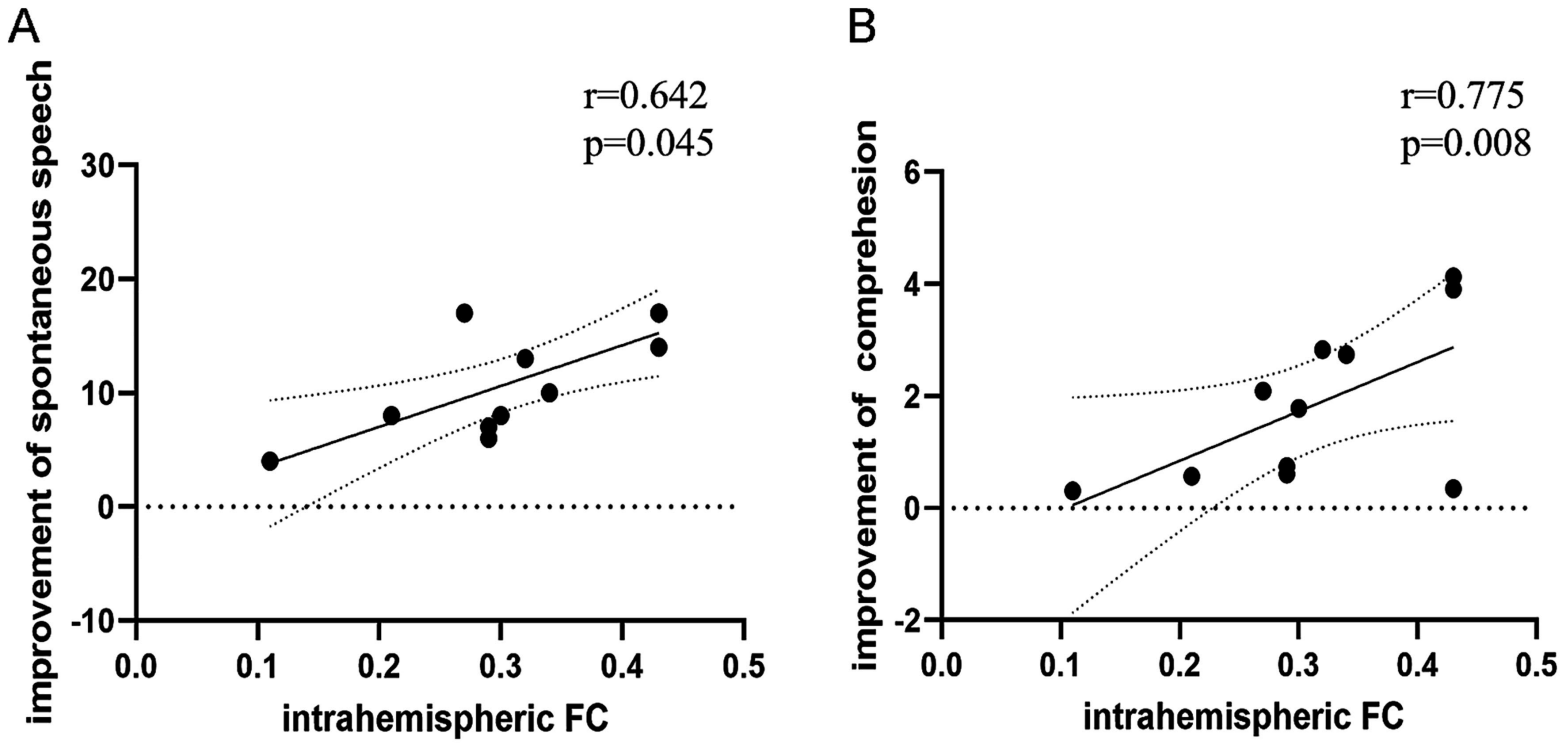
Correlation between baseline functional connectivity (FC) and improvements in language scores from baseline to follow-up. **(A)** Intrahemispheric FC positively correlated with improvement in spontaneous speech (r = 0.642, *p* = 0.045). **(B)** Intrahemispheric FC positively correlated with improvement in auditory comprehension (r = 0.775, *p* = 0.008; two-tailed, uncorrected).

Baseline intrahemispheric FC correlated positively with improvement in spontaneous speech (r = 0.642, *p* = 0.045) and auditory comprehension (r = 0.775, *p* = 0.008) scores from baseline to follow-up (partial correlation analysis; Table 6; Figure 4). No significant associations were found between FC and improvements in other ABC subsections (Table 6), nor between baseline FC and language scores (AQ/subsections) at baseline (Supplementary Table S2) or follow-up (Supplementary Table S3).

**Table 6:** Correlation between baseline functional connectivity (FC) and improvements in language scores from baseline to follow-up in the patients (r, *p*).

The improvement of language performance (*n* = 11)	Intrahemispheric FC	Interhemispheric FC
The improvement of spontaneous speech	(0.642, 0.045^*^)	(0.240, 0.504)
The improvement of auditory comprehension	(0.775, 0.008^**^)	(0.220, 0.542)
The improvement of repetition	(0.437, 0.207)	(0.410, 0.239)
The improvement of naming	(0.374, 0.288)	(0.482, 0.159)
The improvement of AQ	(0.574, 0.082)	(0.506, 0.136)

FC, functional connectivity; AQ, Aphasia quotient. ^*^p < 0.05, ^**^p < 0.01. Statistical analysis: Partial correlations were performed between baseline functional connectivity (FC) and improvements in language scores from baseline to follow-up. *n* = 11 patients who completed language assessments at both T1 and T2.

## 4 Discussion

Rs-fMRI was employed to investigate functional alterations in intrahemispheric and interhemispheric connectivity within the language network of PSA patients and examine correlations between these connectivity patterns and language recovery. Our study suggests that reorganization of residual language network connectivity predicts language recovery of aphasia following left hemisphere lesions.

Our ROIs focused on core language regions—IFG, MFG, and STG—consistent with established language network models (6, 46, 47). While this selection does not represent the entire language system, these regions constitute canonical perisylvian language areas. The IFG is fundamentally associated with language production, with lesions resulting in Broca’s aphasia (48). The STG supports auditory comprehension, while the MFG contributes to integrative language processes (49). Task-based fMRI studies confirm robust activation of the IFG during speech production and the STG during comprehension tasks (43). Using these anatomically and functionally defined ROIs, we generated a normative language network from the HCs, which subsequently enabled the quantification of intrahemispheric and interhemispheric FC alterations in subacute PSA.

Consistent with established theory (50–52), both PSA patients and HCs exhibited stronger intrahemispheric FC than interhemispheric FC within the language network. Crucially, the PSA patients demonstrated significantly enhanced intrahemispheric FC, while interhemispheric FC showed no significant group differences—although a trend toward reduced interhemispheric connectivity was observed in the patients with PSA compared to the HCs. This pattern suggests that post-stroke functional reorganization preferentially engages residual language areas within the left hemisphere, potentially at the expense of interhemispheric integration. Stroke-induced disruption of language networks triggers compensatory reorganization (13, 53–55), with our results indicating early-stage reliance on intrahemispheric pathways.

This finding aligns with longitudinal studies documenting increased left hemisphere connectivity during the acute-to-subacute transition in PSA (56, 57). This interpretation is reinforced by Saur et al.’s (58) task-based fMRI findings, which showed left hemisphere hyperactivation exceeding healthy control levels during the subacute phase. Our prior observation of reduced left IFG–right hemisphere connectivity further supports this model of intrahemispheric compensation (33). These converging findings underscore the pivotal role of the residual left hemisphere region in language rehabilitation. This upregulated intrahemispheric connectivity likely represents a key mechanism underlying early language network adaptation following left hemisphere lesions.

Compared to the healthy controls, the PSA patients exhibited increased intrahemispheric functional connectivity that positively correlated with language recovery—particularly in spontaneous speech and auditory comprehension domains. This pattern likely reflects compensatory reorganization within residual left hemisphere language networks. The dynamic interactions among classical language areas underlie complex linguistic functions (6, 59). This interpretation is substantiated by complementary evidence: Fridriksson et al. (60) demonstrated that increased activation in preserved left hemisphere regions correlates with improved language outcomes in aphasia, while significant associations exist between comprehension recovery and intrinsic connectivity changes within left frontoparietal networks (61). These observations likely reflect upregulated activity within residual language networks, serving as potential predictors of language improvement. Consequently, therapeutic strategies should prioritize modulation of preserved intrahemispheric connectivity as a primary objective for functional recovery.

The primary limitations of this study include its modest sample size and limited follow-up retention. Additional constraints merit consideration: First, our language network was derived from only eight regions of interest, which may inadequately represent the full complexity of post-stroke language reorganization; second, the inclusion of heterogeneous aphasia subtypes without classification analysis; and third, correlation analyses lacked correction for multiple comparisons. Future investigations should validate these findings using larger cohorts, longer follow-up periods, and expanded ROI selections. Furthermore, exploring differential neural mechanisms across aphasia classifications represents a valuable research direction.

## 5 Conclusion

This study demonstrated significantly stronger intrahemispheric than interhemispheric FC in PSA. Furthermore, the PSA patients exhibited increased intrahemispheric FC relative to the HCs. Critically, enhanced intrahemispheric FC correlated positively with language improvement in aphasia. These findings highlight the importance of preserved left hemisphere regions for language recovery after stroke. Elucidating these network mechanisms in subacute PSA offers valuable insights for developing targeted rehabilitation strategies and informing individualized therapeutic interventions.

## Data Availability

The raw data supporting the conclusions of this article will be made available by the authors, without undue reservation.
