# Supplementary material for: Relationship between intrahemispheric and interhemispheric connectivity of the language network and language improvement in subacute post-stroke aphasia
Source: Front Neurol. 2025 Dec 12;16:1634902. doi: 10.3389/fneur.2025.1634902 (PMC12740746; doi:10.3389/fneur.2025.1634902)
Supplement: Supplementary file 1 [file Data_Sheet_1.pdf]

Supplementary Material

Table S1 Lesion Topography, Aphasia Classification, and Aphasia Quotient Scores in Patients with Aphasia

ID	Lesion Location	Type of Aphasia	AQ Score
001	Left temporal lobe, parietal lobe, occipital lobe	Wernicke aphasia	30.86
002	Left frontal lobe	Broca aphasia	11.74
003	Left paraventricular white matter	Broca aphasia	10.78
004	Left parietal lobe	Broca aphasia	33.24
005	Left frontal lobe, temporal lobe	Conduction aphasia	61.38
006	Left temporoparietal junction	Anomic aphasia	88.52
007	Left frontal lobe, temporal lobe, parietal lobe	Global aphasia	7.3
008	Left frontal lobe, insula	Broca aphasia	12.87
019	Left temporal lobe, occipital lobe	Global aphasia	7.3
010	Left frontal lobe,	Wernicke aphasia	26.72
011	Left temporal lobe, temporoparietal junction	Wernicke aphasia	43.8
012	Left frontal lobe, insula	Wernicke aphasia	50.13
013	Left temporal lobe,	Anomic aphasia	78.18
014	Left frontal lobe	Anomic aphasia	91.06
015	Left temporal lobe, temporoparietal junction	Global aphasia	15.98
016	Left temporal lobe, occipital lobe	Wernicke aphasia	47.1
017	Left centrum semiovale	Anomic aphasia	78.12
018	Left temporal lobe	Wernicke aphasia	18
019	Left frontal lobe, parietal lobe	Wernicke aphasia	56.44
020	Left frontal lobe, parietal lobe, centrum semiovale	Broca aphasia	25.7
021	Left paraventricular white matter, basal ganglia	Anomic aphasia	82.57
022	Left temporal lobe, parietal lobe	Conduction aphasia	70.29
023	Left frontal lobe, basal ganglia	Wernicke aphasia	45
024	Left paraventricular white matter, basal ganglia	Anomic aphasia	72.25
025	Left frontal lobe, parietal lobe	Broca aphasia	41.34
026	Left centrum semiovale, paraventricular white matter	Global aphasia	14.03
027	Left temporal lobe, insula, paraventricular white matter	Global aphasia	0.2
028	Left centrum semiovale,	Broca aphasia	14.66

	paraventricular white matter, insula, basal ganglia		
029	Left paraventricular white matter, parietal lobe	Anomic aphasia	58.74
030	Left insula, parietal lobe	Global aphasia	19.95
031	Left parietal lobe, paraventricular white matter	Global aphasia	33.19
032	Left paraventricular white matter, basal ganglia	Broca aphasia	34.92

Note: AQ, Aphasia quotient.

Table S2 Correlation Between Functional Connectivity (FC) and Baseline Language Scores in Patients (r, p)

n =32	Intrahemispheric FC	Interhemispheric FC
Spontaneous speech	(-0.108, 0.556)	(-0.124, 0.499)
Auditory comprehension	(-0.152, 0.405)	(-0.117, 0.523)
Repetition	(-0.183, 0.316)	(-0.153, 0.402)
Naming	(-0.115, 0.529)	(-0.189, 0.299)
AQ	(-0.165, 0.368)	(-0.150, 0.411)

Note: FC, functional connectivity; AQ, Aphasia quotient.

Table S3 Correlation Between Baseline Functional Connectivity (FC) and Follow-up Language Scores in Patients (r, p)

n =11	Intrahemispheric FC	Interhemispheric FC
Spontaneous speech	(0.244, 0.496)	(0.111, 0.760)
Auditory comprehension	(0.276, 0.440)	(0.572, 0.084)
Repetition	(-0.078, 0.830)	(0.106, 0.770)
Naming	(0.168, 0.643)	(0.427, 0.218)
AQ	(0.099, 0.785)	(0.390, 0.265)

Note: FC, functional connectivity; AQ, Aphasia quotient.
